# Supplementary material for: Nanoscale size effects in α-FAPbI3 evinced by large-scale ab initio simulations
Source: Nat Commun. 2025 Jul 24;16:6825. doi: 10.1038/s41467-025-61351-7 (PMC12289870; doi:10.1038/s41467-025-61351-7)
Supplement: Supplementary file 1 — Supplementary Information [file 41467_2025_61351_MOESM1_ESM.pdf]

Supplementary Information

Nanoscale size effects in α-$FAPbI_3$ evinced by large-scale ab initio simulations

Virginia Carnevali,[†,‡] Lorenzo Agosta,[†,‡] Vladislav Slama,[†] Nikolaos Lempesis,[†,#] Andrea Vezzosi,[†] and Ursula Rothlisberger[†,*]

†Laboratory of Computational Chemistry and Biochemistry, Institute of Chemical Sciences and Engineering, Swiss Federal Institute of Technology (EPFL), Lausanne, Switzerland

‡these authors contributed equally to this work

#Current address: Department of Chemistry, University of Ioannina, Ioannina 45110 Greece

E-mail: ursula.roethlisberger@epfl.ch

Supplementary Note 1. FA 3-fold symmetry

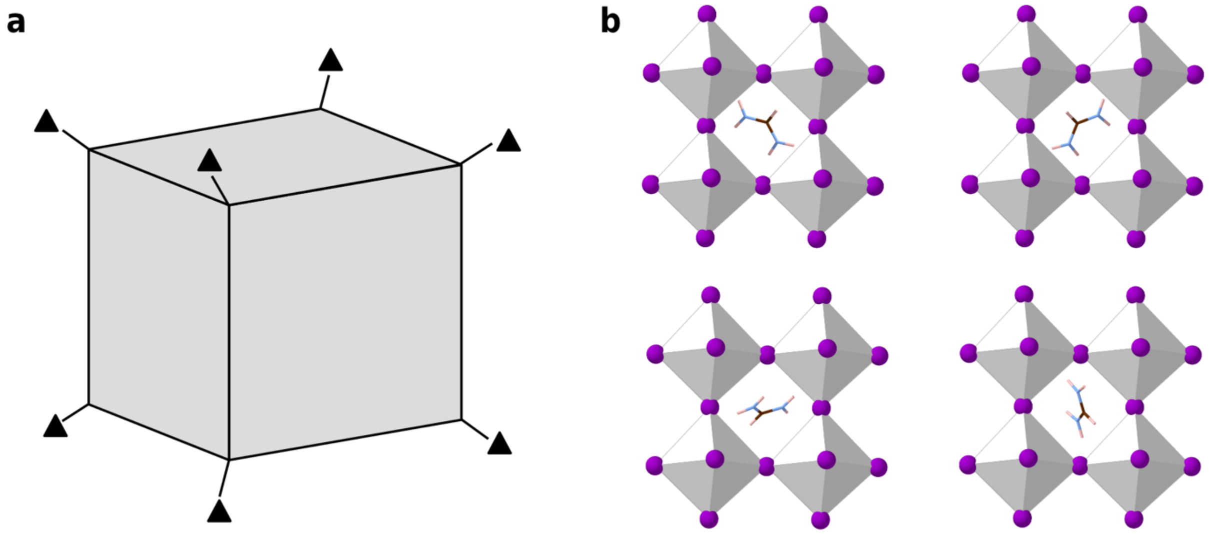

Supplementary Figure 1: FA 3-fold symmetry. (a) The four 3-fold axes of the cubic symmetry. (b) Orientations of the FA molecule such that its molecular dipole satisfies the 3-fold symmetry within the PbI_6 cage. I, Pb, C, N, and H are shown in violet, white octahedra, brown, light blue, and pink, respectively.

Supplementary Note 2. Octahedra tilting

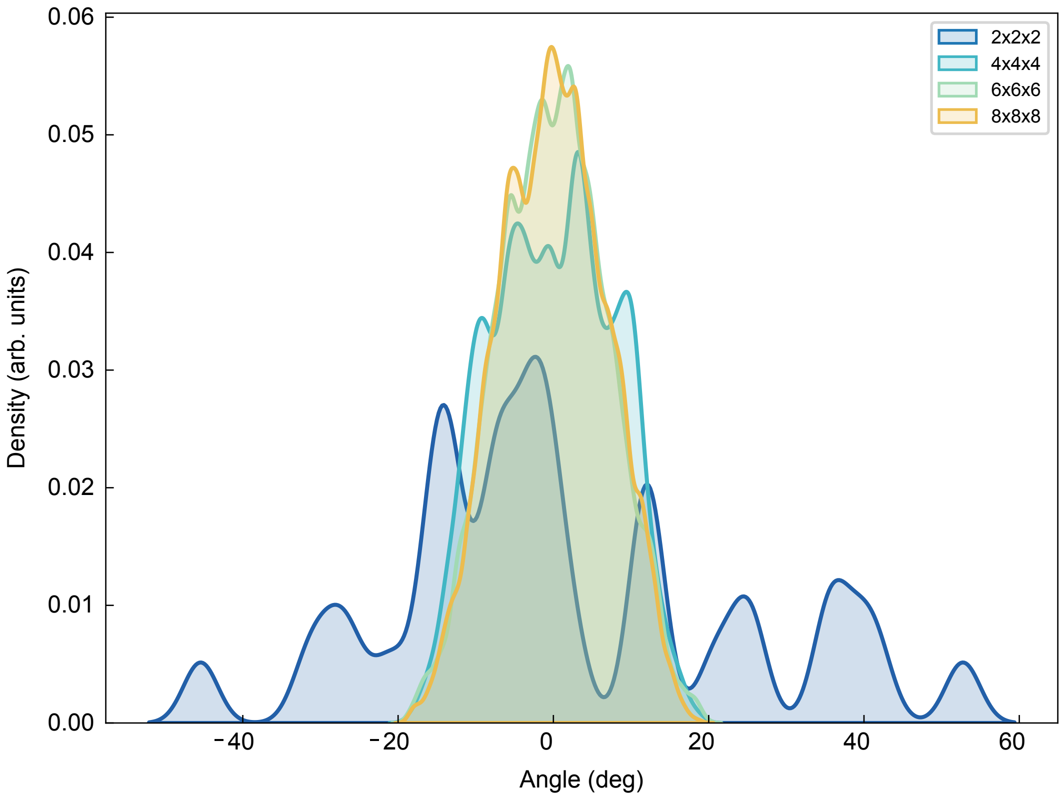

Supplementary Figure 2: Octahedra tilting. Distributions of the octahedra tilting angles for the different supercells optimized from a pseudo-random FA configuration. Source data are provided as zip folder SourceData_FigS2.

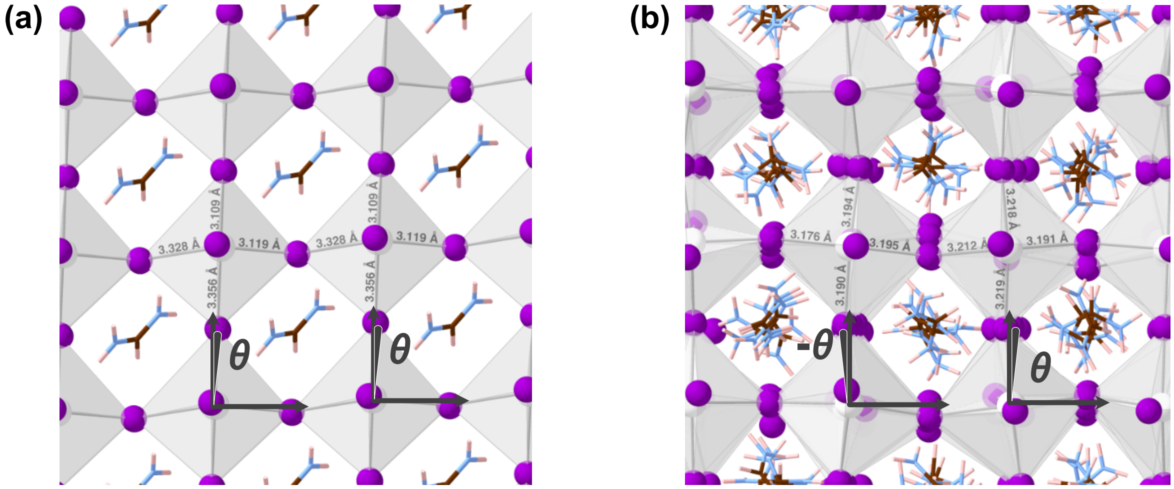

Supplementary Figure 3: All-aligned versus pseudo-random FA induced octahedra tilting. Zoom of the final $FAPbI_3$ structures starting from all-aligned (a) and pseudo-random (b) FA configurations after vc-relax. The Pb-I distances for two adjacent octahedra are highlighted. The typical $-\theta/\theta$ tilting pattern of the octahedra can be seen in the pseudo-random case, while the all-aligned case shows a non-physical collective I-shift resulting in a tilting angle of θ for all octahedra. The reference system for the tilt angle and the tilting angles are shown in red. The color code is the same as in Fig.1.

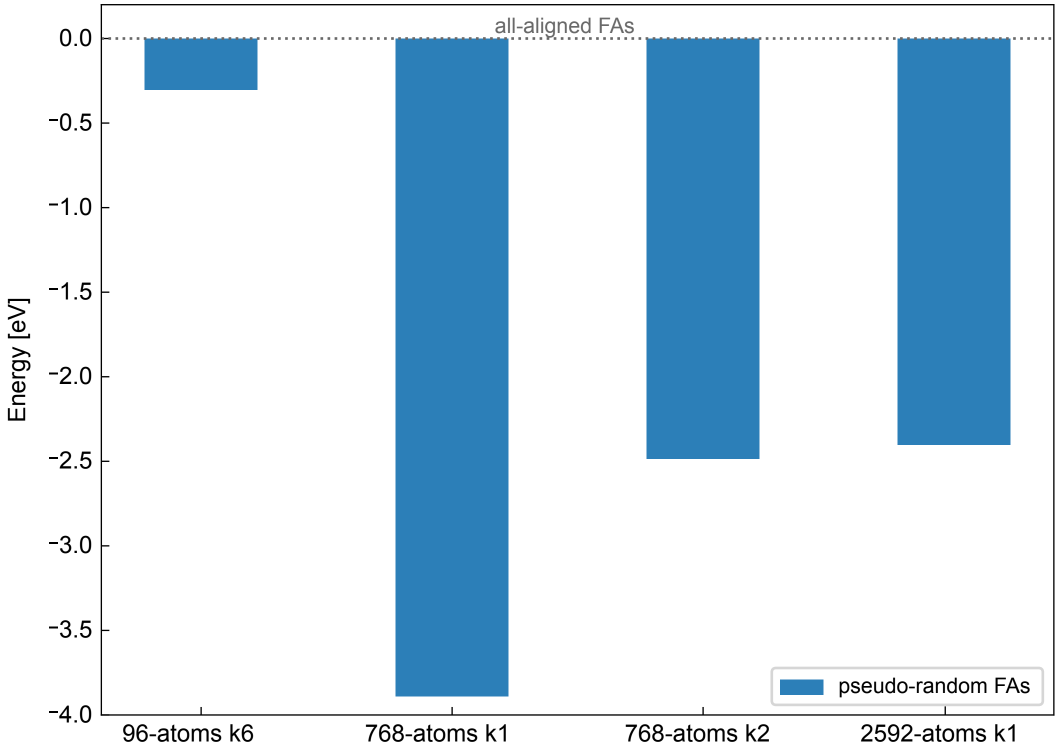

Supplementary Figure 4: All-aligned versus pseudo-random FA energetic. Comparison of the total energy per unit cell between the all-aligned and pseudo-randomly oriented FA configurations for different supercells. On the x-axis, the number of atoms in the supercell and the k-point grid (e.g., k1=1×1×1 k-point grid) are given. The all-aligned FA configurations (gray dotted line) are always higher in energy (less stable) and are set to 0 eV for each supercell. The energies of the pseudo-randomly oriented FA configurations (blue) are given with respect to the corresponding value of the all-aligned ones. Source data are provided as zip folder SourceData_FigS4.

Supplementary Note 3. Band gap

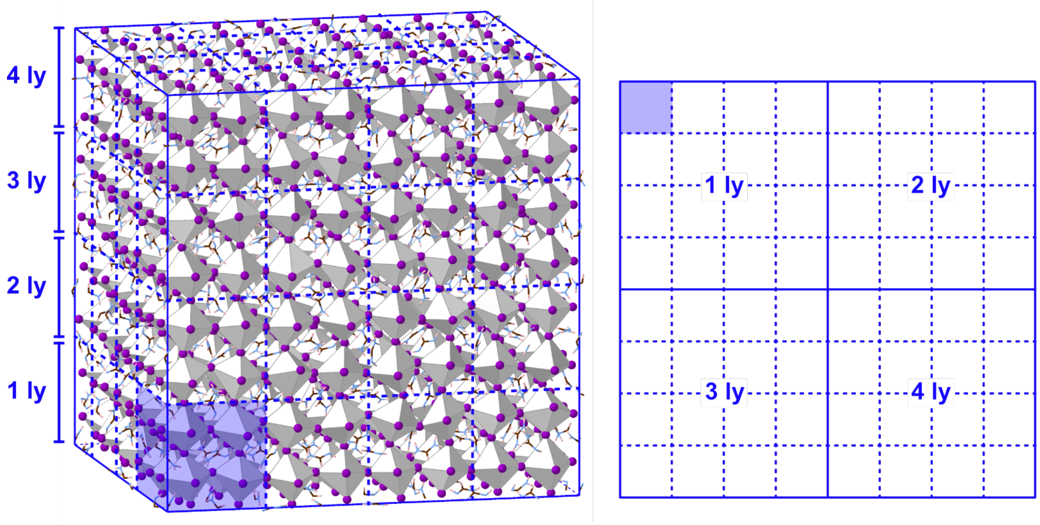

Supplementary Figure 5: Schematic of 6x6x6 supercell splitting into 2x2x2 supercells. Band gap mapping of the 6144-cell from 3D to 2D. 64 96-cells were identified in the 6144-cell, as shown by the blue grid superimposed on the 3D model in the left panel, and rearranged into a 2D map as shown in the right panel. The area shaded in blue highlights a single 96-cell. The colour code is the same as in Fig.1.

Supplementary Table 1: Valence and conduction bands edges. $FAPbI_3$ Kohn-Sham valence band maximum (VBM) and conduction band minimum (CBM) for different system sizes at several theory levels and system optimization schemes. The initial configurations were chosen with all FAs aligned except the ones indicated with a star superscript. Source data are provided as SourceData_TableS1.txt.

Simulation cell	relax PBE 0 K (eV)		vc-relax PBE 0 K (eV)		vc-relax PBE+SOC 0 K (eV)		vc-relax PBE0 0 K (eV)		vc-relax PBE0+SOC 0 K (eV)		$n \times n \times n$ k-point grid
	VBM	CBM	VBM	CBM	VBM	CBM	VBM	CBM	VBM	CBM	
12-atoms $(1 \times 1 \times 1)$	1.70	5.38	2.61	6.33	2.86	6.32	1.53	7.35	1.80	7.34	1
	2.31	4.51	1.90	4.20	2.02	3.79	0.90	4.81	1.04	4.37	2
	2.53	4.02	1.81	3.78	1.94	3.18	0.89	4.35	1.00	3.72	4
	2.53	4.03	2.30	3.93	2.50	2.99	1.18	4.62	1.40	3.68	6
	2.53	4.03	2.31	3.89	2.51	2.99	1.19	4.57	1.41	3.66	8
	2.53	4.02	2.31	3.86	2.51	2.96	1.19	4.56	1.41	3.66	10
96-atoms $(2 \times 2 \times 2)$	2.30	4.54	2.31	4.88	2.09	3.88	1.25	4.61	1.38	4.24	1
	2.49	4.04	2.22	3.91	2.41	3.02	1.42	4.27	1.63	3.27	2
	2.53	4.02	2.30	3.89	2.51	3.00	1.51	4.24	-	-	4
	2.54	4.02	2.28	3.89	2.48	3.02	1.67	4.42	-	-	6
	2.45*	4.06*	2.46*	4.06*	2.65*	3.13*	-*	-*	-*	-*	6
768-atoms $(4 \times 4 \times 4)$	2.50	4.04	2.22	3.83	2.44	2.92	-	-	-	-	1
	2.38*	4.12*	2.38*	4.12*	2.57*	3.20*	-*	-*	-*	-*	1
	2.31	3.88	2.31	3.88	2.51	2.96	-	-	-	-	2
	2.42*	4.11*	2.42*	4.11*	2.61*	3.17*	-*	-*	-*	-*	2

* FA pseudo-randomly oriented

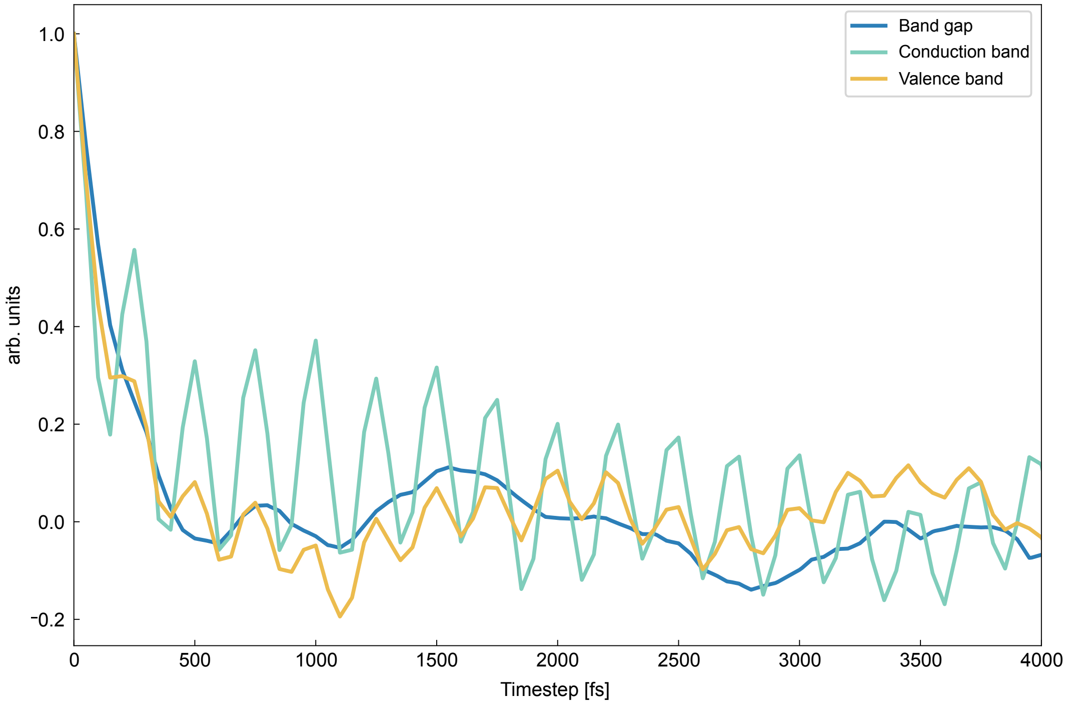

Supplementary Figure 6: Band gap, valence, and conduction bands correlations. Time correlation function characterizing the band gap oscillations (blue curve), the bottom of the conduction band eigenvalue (orange curve), and the top of the valence band eigenvalue (green curve). The analysis was done on the 2592-atom one. Source data are provided as zip folder SourceData_FigS6.

Supplementary Note 4. Dipole moment

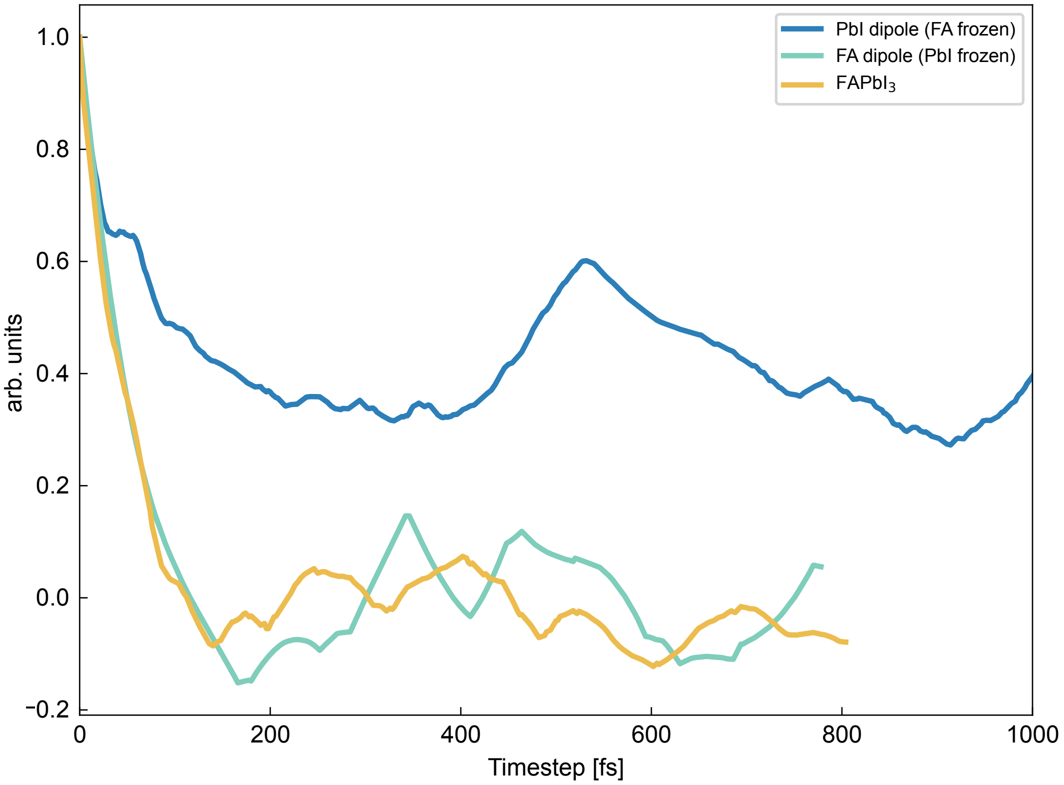

Supplementary Figure 7: Band gap, PbI, and FA dipole correlations. Time correlation function characterizing the dipole moment oscillations of the PbI_6 (blue curve), the FAs (orange curve), and the total one (green curve) for the 768-atom cell. Two NPT-F simulations were performed with FA or PbI_6 frozen to get the two separate contributions - PbI_6 and FA - to the dipole moment. FA alone cannot compensate the dipole moment of the lattice, but the PbI_6 fluctuations can (correlation goes to 0). Source data are provided as zip folder SourceData_FigS7.

Fig.7 shows the different starting configurations of FAs for the 768-atom cell. In the "aligned" configuration all the FAs are aligned in the same direction, in the "random" configuration they are oriented randomly, in the "random_best" configuration they are oriented randomly so as to minimize the dipole moment, in the "smart_100" configuration they are oriented along the 100, 010, 001 directions minimizing the dipole moment, in the "smart_quasi" configuration they are pseudo-randomly oriented preserving the 3-fold symmetry (Supplementary Fig.1) and minimizing the dipole moment.

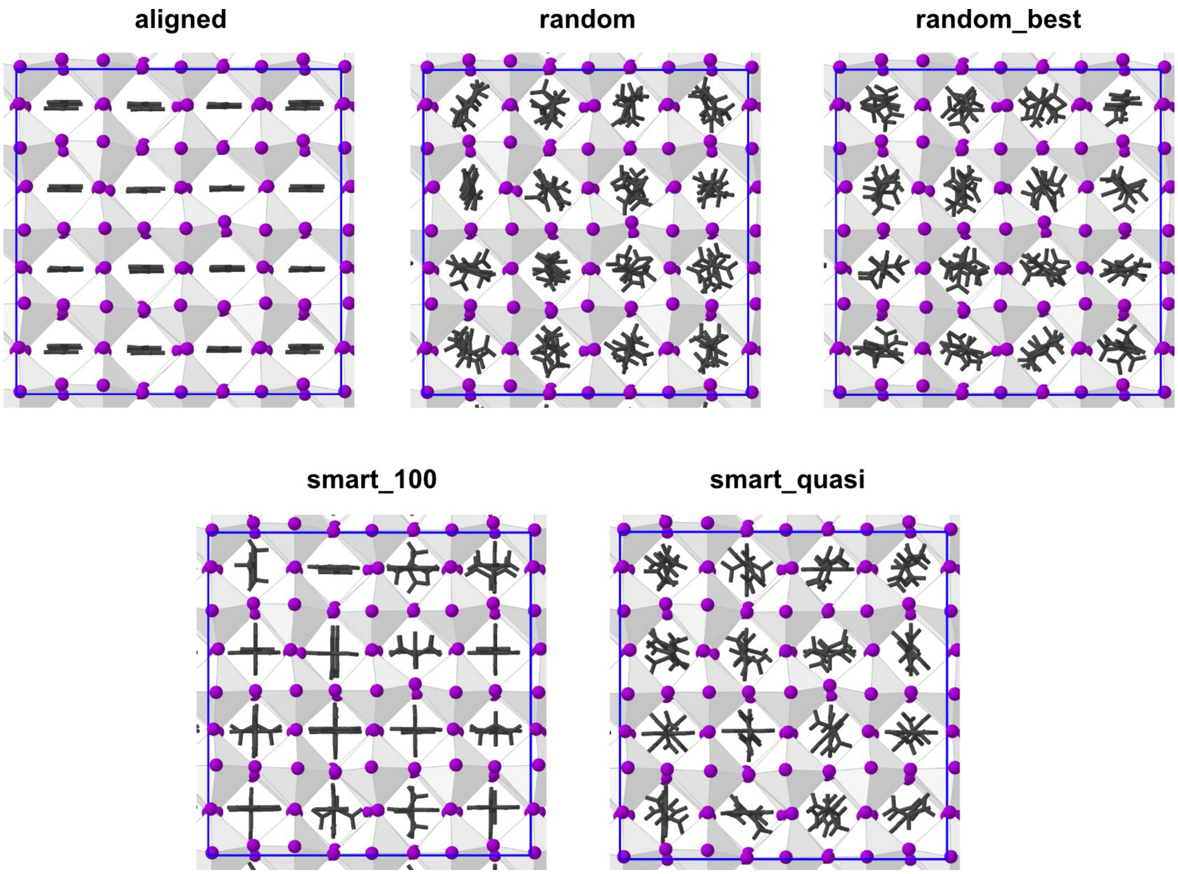

Supplementary Figure 8: Different initial FA configurations. Initial configurations of the 768-atom cell with different FA orientations. FA molecules are shown in dark grey to facilitate the visualization. I and Pb are shown in violet and white octahedra, respectively.

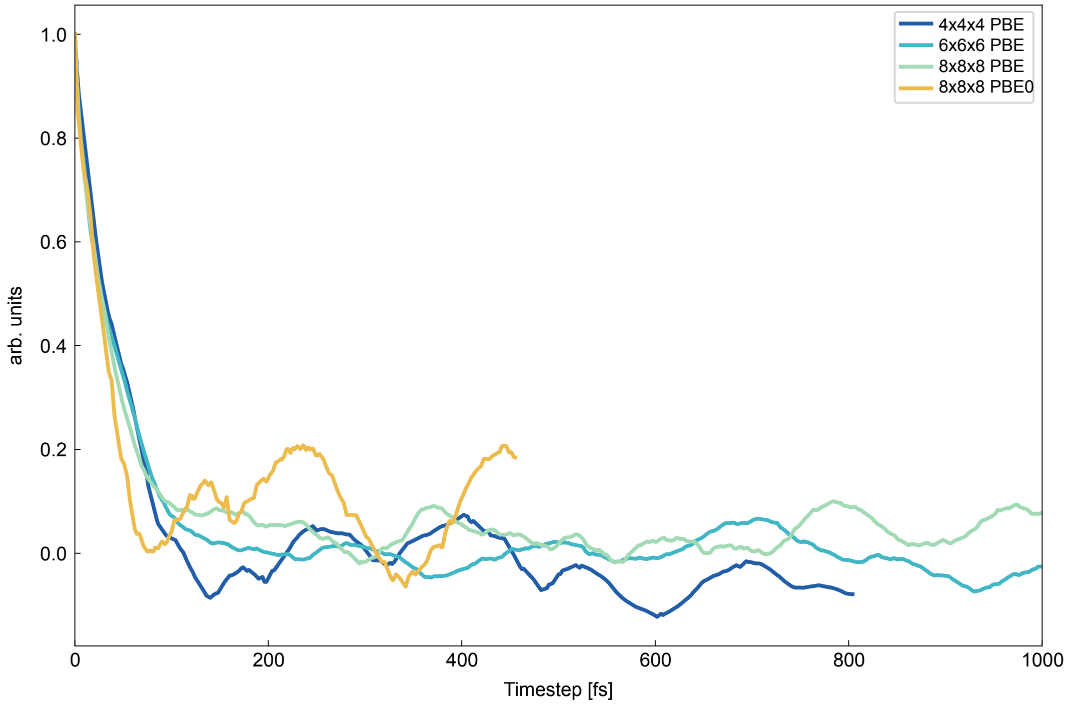

Supplementary Figure 9: Dipole correlation versus supercell size. Time correlation function characterizing the dipole moment oscillations for different supercell size. The 6144-atom cell results are reported with both PBE and PBE0 level of theory. Source data are provided as zip folder SourceData_FigS9.
